# Supplementary material for: TBX21 Methylation as a Potential Regulator of Immune Suppression in CMS1 Subtype Colorectal Cancer
Source: Cancers (Basel). 2022 Sep 22;14(19):4594. doi: 10.3390/cancers14194594 (PMC9558549; doi:10.3390/cancers14194594)
Supplement: Supplementary file 1 [file cancers-14-04594-s001.zip › cancers-1768880_SupplementaryFiles/cancers-1768880_SupplementaryFigures.pdf]

Figure S1

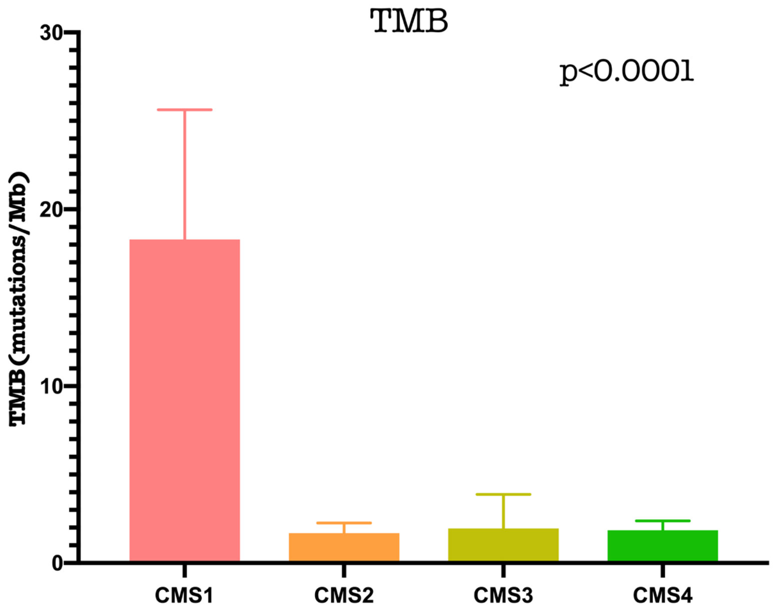

Figure S1. Bar chart to show the level of TMB in each CMS subtype. CMS1 patients showed significantly higher TMB.

Figure S2A

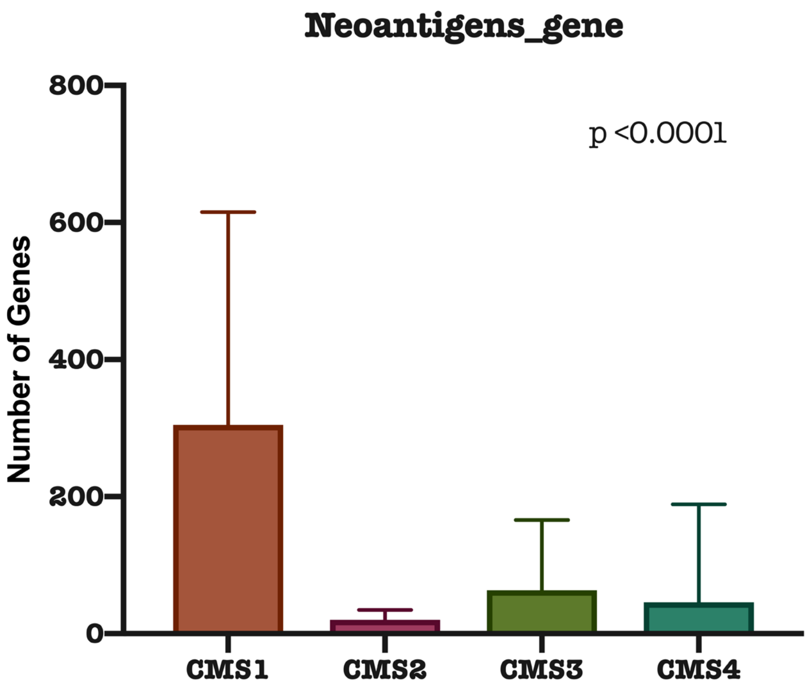

Figure S2B

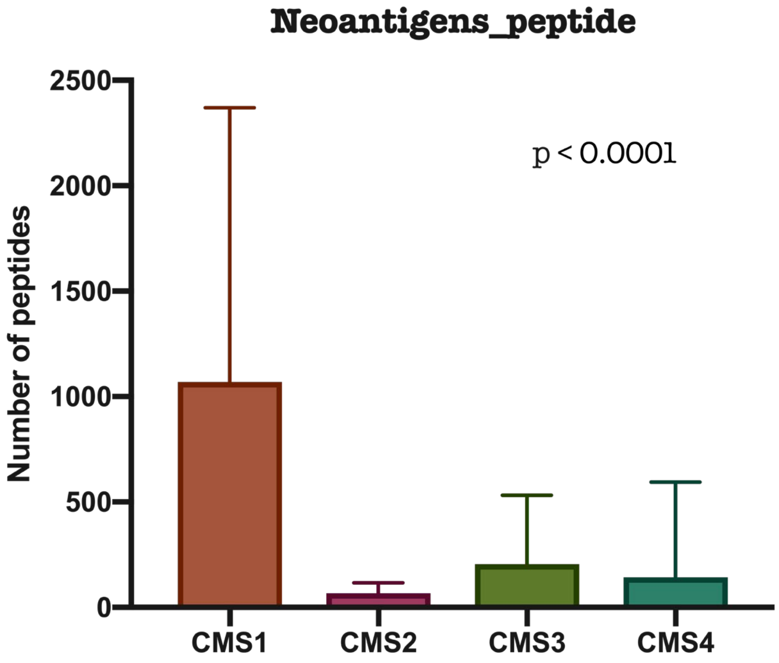

Figure S2. Bar chart to show the level of neoantigens associated: A. genes; B. neopeptides. CMS1 patients showed significantly higher neoantigens.
